# Prognostic value of the Hippo pathway transcriptional coactivators YAP/TAZ and β1-integrin in conventional osteosarcoma

**DOI:** 10.18632/oncotarget.11876

**Published:** 2016-09-06

**Authors:** Corinne Bouvier, Nicolas Macagno, Quy Nguyen, Anderson Loundou, Carine Jiguet-Jiglaire, Jean-Claude Gentet, Jean-Luc Jouve, Alexandre Rochwerger, Jean-Camille Mattei, Daniel Bouvard, Sébastien Salas

**Affiliations:** ^1^ Aix-Marseille University (AMU), Faculty of Medecine, CRO2, UMR 911 (Equipe IV), Marseille, France; ^2^ Department of Pathology, APHM, Timone Hospital, Marseille, France; ^3^ Department of Public Health, Aix-Marseille University (AMU), Faculty of Medecine, EA 3270 Research Unit, Marseille, France; ^4^ Department of Research and Innovation, APHM, Timone Hospital, Support Unit for Clinical Research and Economic Evaluation, Marseille, France; ^5^ Department of Pediatric Oncology, APHM, Timone Hospital, Marseille, France; ^6^ Department of Pediatric Orthopaedic Surgery, APHM, Timone Hospital, Marseille, France; ^7^ Department of Adult Orthopaedic Surgery, APHM, Nord Hospital, Marseille, France; ^8^ Institut Albert Bonniot, U823, Grenoble, France

**Keywords:** osteosarcoma, hippo pathway, YAP/TAZ, beta1 integrin, prognosis, Pathology Section

## Abstract

**Introduction:**

Currently, very few studies are available concerning the mammalian Hippo pathway in bone sarcomas. YAP/TAZ transcription co-activators are key downstream effectors of this pathway and may also have oncogenic properties. Additionally, recent *in-vitro* experiments showed that expression of β1-integrin promoted metastasis in osteosarcomas. This study investigated the expression of YAP/TAZ and β1-integrin in human osteosarcomas.

**Materials and methods:**

We performed automated immunohistochemistry on tissue-microarrays (TMA) in which 69 conventional osteosarcomas biopsies performed prior to chemotherapy were embedded. Cellular localization and semi-quantitative analysis of each immunostain was performed using Immunoreactive Score (IRS) and correlated to clinico-pathological data.

**Results:**

Cytoplasmic expression of β1-integrin was noted in 54/59 osteosarcomas (92%), with 33/59 cases (56%) displaying membranous staining. YAP/TAZ was expressed in 27/45 osteosarcomas (60%), with 14 cases (31%) showing cytoplasmic expression while 13 other cases (28%) displayed nuclear expression. No link was found between YAP/TAZ or β1-integrin expression and response to chemotherapy. In univariate analysis, YAP/TAZ immunoreactive score was pejoratively correlated with overall survival (*p* = 0.01). Expression of β1-integrin on cell membrane was also pejorative for OS (*p* = 0.045). In multivariate analysis, YAP/TAZ nuclear expression was an independent prognostic factor for PFS (*p* = 0.035).

**Conclusion:**

this study indicates that β1-integrin and YAP/TAZ proteins are linked to prognosis and therefore could be therapeutic targets in conventional osteosarcomas.

## INTRODUCTION

Conventional osteosarcoma is the most frequent primary bone tumor: it is a high-grade sarcoma with frequent metastases. Following the introduction of neoadjuvant chemotherapy, prognosis of conventional osteosarcoma improved dramatically, leading to a 5-year overall survival rate of 50-70%. For some patients, however, this treatment is still insufficiently effective: patients may develop metastatic disease or become refractory to chemotherapy. Identification of new potentially drugable targets related to osteosarcoma tumorigenesis is therefore crucial to improve the treatment of this neoplasm.

YAP (Yes-Associated Protein) and TAZ (Transcriptional coactivator with PDZ-binding motif) are downstream effectors of the hippo pathway and serves as transcriptional co-activators. This pathway is well conserved during evolution probably owing to its involvement in development: the hippo pathway is necessary to regulate the size of organs, tissue homeostasis and tissue repair in mammals and drosophila [1]. The binding of YAP/TAZ along with other transcriptional factors on specific sequences (which are called TEA domain-containing sequence-specific transcription factors, namely TEADs) induces proliferation, self-renewal, differentiation and survival of the cells [2].

Experiments have suggested a potential oncogenic role of YAP: YAP expression induced epithelial-mesenchymal transition, abolished apoptosis and promoted proliferation [3]. YAP expression is also linked to oncogenic properties in several human malignancies such as hepatocellular carcinoma, non-small cell lung carcinoma, breast carcinoma, esophageal squamous cell carcinoma, ovarian and gastric cancers [4-9]. While biology of the Hippo pathway was mainly described in epithelial malignancies, only scarce reports have highlighted its potential role in sarcomas [10]. YAP knockdown in Ewing's sarcoma cells inhibited cell proliferation and anchorage-independent colony formation [11]. TAZ has been identified in bone as a transcriptional co-activator of RUNX2 during osteogenic differentiation [10].

Recently, some reports showed that aberrations in Hippo Pathway may be important events in the biology of osteosarcomas [12-14]: YAP suppression *in vitro* on OS cell lines (osteosarcoma-derived cell lines) was associated with a decrease in both proliferation and invasion. *In vivo,* decreased tumor growth was also observed with YAP suppression in OS cell lines murine xenografts and transgenic mice. Zhang *et al*. reported higher expression of YAP1 in osteosarcomas compared to non-cancerous tissues, YAP expression being also correlated with Ennekin Staging System, albeit not correlated with other clinical parameters such as age, location and metastases [14].

Integrins play a role in tumor growth and metastasis [15]. Previous studies have shown the presence of β1-integrin on the cellular membrane processes of SAOS-2 and MG63 osteosarcoma cells [16]. The use of AIIB2 antibody, an anti-β1-integrin monoclonal antibody, was reported to greatly inhibit the seeding of osteosarcoma cells on the lung *in vitro* [17]. Recently, β1-integrin was thought to play a role in the YAP/TAZ signaling axis: in mesenchymal progenitors, the membrane-anchored metalloproteinase MT1-MMP could regulate stem cells shape by activating a β1-integrin /Rho-GTPase signaling cascade and triggering the nuclear location of YAP/TAZ [18].

To explore the Hippo signaling pathway in osteosarcomas, we performed an immunohistochemical study with anti-YAP/TAZ and anti-β1-integrin antibodies on 69 high-grade osteosarcomas biopsies. We correlated immunohistochemical protein expression with clinical parameters such as chemotherapy response, progression-free survival (PFS) and overall survival (OS). We found that YAP/TAZ and β1-integrin expression both had a prognostic value.

## RESULTS

### Patients characteristics

The clinico-pathological characteristics of the 69 patients are summarized in Table 1. Sex ratio was 1,3:1 and the median of age was 13.9 years. All tumors were located in long bones with a mean tumor size of 11.72 cm (2.5-34 cm).

**Table 1:** clinical data of the 69 patients

Sex-ratio	30 females	(43.5%)
	39 males	(56.5%)
Median age	13.9 years	(9 months - 70.4 years)
Response to preoperative chemotherapy	33 good responders	(48%)
	33 bad responders	(48%)
	3 unknown	(4%)
Tumor location	60 cases lower limb	(87%)
	9 cases upper limb	(13%)
Mean tumor size	11.72 cm	(2.5 – 34 cm)
Median follow-up	45 months	(6 months – 14.4 years)
Deaths during follow-up	16 patients	(23.2%)
Metastatic evolution	23 patients	(33%)
Median recurrence time	36 months	(2 months – 14 years)

* good responders correspond to inferior or equal to 10% of viable tumor after chemotherapy

### Treatment characteristics and outcome

All patients underwent surgical excision after preoperative conventional chemotherapy (OS94 and OS06 regimens). After pathological examination of the post-chemotherapy specimen, 33 patients were considered good responders and 33 patients considered bad responders to chemotherapy, response to chemotherapy data were not available for 3 patients. Median of follow up was 45 months (0.5-14.4 years), 16 patients (23,2%) died during the follow-up and 23 patients (33%) developed metastases. Median time of recurrence was 3 years.

**Table 2:** immunohistochemical data for β1-integrin

	β1-Integrin expression	
**Negative**	**5/59**	**9%**
**Positive**	**54/59**	**92%**
Strictly cytoplasmic	54/59	92%
Cytoplasmic and membranous	33/59	56%

### β1-integrin and YAP/TAZ expression in biopsies of osteosarcomas

#### Pattern of staining and IRS

**Figure 1:**
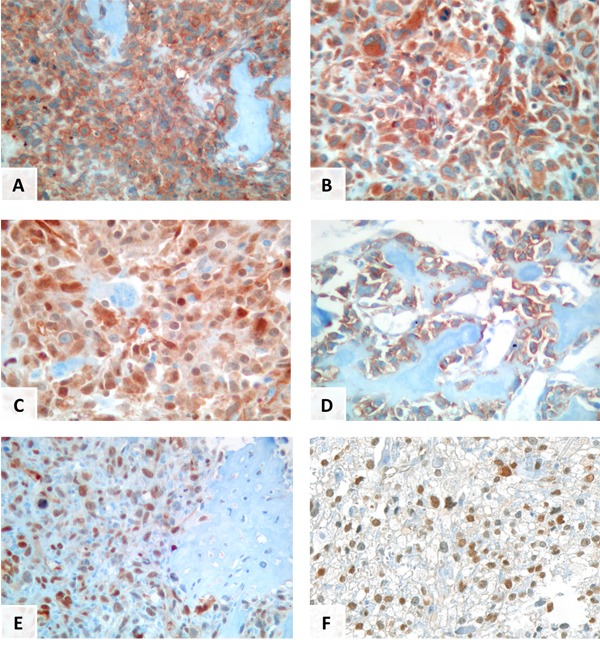
immunostaining patterns with YAP/TAZ and β1-integrin in primary or metastatic conventional osteosarcomas β1-integrin cytoplasmic and membranous staining **a.**, β1-integrin cytoplasmic staining **b.**, YAP/TAZ nuclear and cytoplasmic staining **c.**, YAP/TAZ cytoplasmic staining **d.**, YAP/TAZ nuclear staining **e.**, YAP/TAZ nuclear expression in metastases of conventional osteosarcomas **f.**

Immunochemical results for β1-integrin and YAP/TAZ are summarized in Table 2 and Table 3, respectively. β1-integrin was expressed in the cytoplasm of the tumor cells in 54/59 cases (91.5%) with 33 cases (56%) displaying additionally a membranous positivity (Figure 1a and 1b). YAP/TAZ IHC was positive in 27/45 cases (60%), with an expression in both the cytoplasm and the nucleus in 8 cases (17%, Figure 1c), with strict cytoplasmic expression in 14 cases (31%, Figure 1d) and with strict nuclear expression in 5 cases (11%)(Figure 1e). Semi-quantitative analysis was then performed using IRS: 16 cases were completely negative, 24 showed low/moderate positivity and 5 showed high positivity. IRS of β1-integrin and YAP/TAZ were statistically correlated (*p* = 0.002). Nuclear location of YAP/TAZ was not statistically correlated to β1-integrin membranous immunostaining (*p* = 0.294).

**Table 3:** immunohistochemical data for YAP/TAZ

	YAP / TAZ expression	
**Negative**	**18/45**	**40%**
**Positive**	**27/45**	**60%**
Strictly cytoplasmic	14/45	31%
Strictly nuclear	5/45	11%
Cytoplasmic and nuclear	8/45	17%

#### Prognostic value of β1-integrin and YAP/TAZ expression

In univariate analysis (Table 4), response to chemotherapy had a prognostic value for both PFS (*p* = 0.027) and OS (*p* = 0.015). Two classes YAP/TAZ IRS was correlated with OS (*p* = 0.01). Nuclear location of YAP/TAZ was not statistically correlated with OS but there was a trend to significance with PFS (*p* = 0.112). Membranous expression of β1-integrin was correlated with poor OS (*p* = 0.045).

**Table 4:** univariate analysis

	**PFS**	**OS**
Response to chemotherapy	*P* = 0.027*	*P* = 0.015*
YAP/TAZ IRS	*P* = 0.094	*P* = 0.01*
Nuclear YAP/TAZ expression	*P* = 0.112	*P* = 0.953
Membranous β1-integrin expression	*P* = 0.260	*P* = 0.045*

PFS = progression free survival, OS = overall survival, IRS = Immunoreactive Score (two classes IRS 0-6 versus 7-12)

* statistically significant p value.

In multivariate analysis (Table 5 and Table 6), only YAP/TAZ nuclear expression was an independent prognostic factor for PFS (*p* = 0,035, HR = 4,2, IC 1.11-16.2).

**Table 5:** multivariate analysis with YAP/TAZ IRS

	PFS			OS		
	HR	CI	*P* value	HR	CI	*P* value
Response to chemotherapy	1.671	0.360-7.747	0.512	3.140	0.950-10.377	0.061
YAP/TAZ IRS	0.213	0.035-1.288	0.092	0.173	0.028-1.082	0.61
Membranous β1-integrin expression	0.252	0.028-2.254	0.277	1.063	0.306-3.701	0.923

PFS = progression free survival, OS = overall survival, IRS = Immunoreactive Score (two classes IRS 0-6 *versus* 7-12), HR = hazard ratio, CI = confidence interval

* statistically significant *p* value.

**Table 6:** multivariate analysis with YAP/TAZ nuclear expression

	PFS			OS		
	HR	CI	*p* value	HR	CI	*p* value
Response to chemotherapy	2.994	0.894-10	0.075	0.524	0.117-2.34	0.398
Nuclear YAP/TAZ expression	4.243	1.11- 16.2	0.035*	1.992	0.359-11.05	0.43
Membranous β1-integrin expression	1.905	0.526-6.905	0.327	0.150	0.17-1.37	0.092

PFS = progression free survival, OS = overall survival, IRS = Immunoreactive Score (two classes IRS 0-6 *versus* 7-12), HR = hazard ratio, CI = confidence interval

* statistically significant *p* value.

### β1-integrin and YAP/TAZ expression in metastases

Twenty-three patients developed metastases, and 19 specimens of pulmonary metastases were available: all the cases showed immunohistochemical membranous β1-integrin expression. Most of the cases (16/19, 84%) showed nuclear YAP/TAZ immunostaining and a high IRS (Figure 1f). Compared to biopsy specimens, metastases showed more frequently β1-integrin membranous expression (*p* = 0.004), higher IRS for YAP/TAZ (*p* < 0.0083) and YAP/TAZ nuclear staining was more frequent (*p* < 0.0011).

### β1-integrin and YAP/TAZ expression and correlation with response to chemotherapy

No statistically significant correlation was found between YAP/TAZ IRS or β1-integrin IRS and response to chemotherapy nor between nuclear YAP/TAZ or membranous β1-integrin immunostainings and response to chemotherapy.

**Figure 2:**
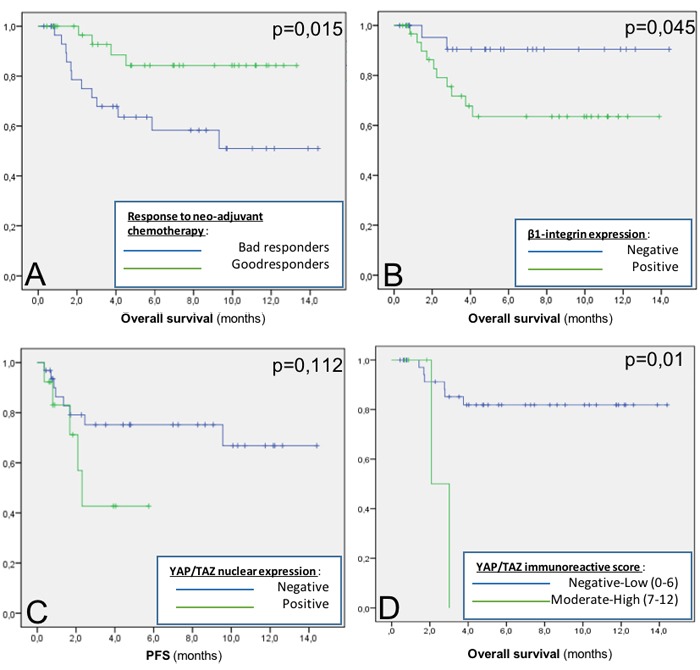
Overall Survival (OS) and Progression Free Survival (PFS) OS according to neo-adjuvant therapy response **a.**, OS according to β1-integrin expression **b.**, PFS according to YAP/TAZ nuclear expression **c.**, OS according to YAP/TAZ IRS **d.**

## DISCUSSION

Few years ago it was suggested that the Hippo signaling pathway might play a role in osteogenic differentiation: expression of an active TAZ mutant enhanced RUNX2-driven gene expression [22, 23] while knockdown of TAZ in mesenchymal stem cells (MSCs) inhibited osteogenesis when the cells were cultured under conditions favoring osteoblast differentiation [22]. Conversely, when an activated YAP mutant was overexpressed in MSCs, osteogenic differentiation was promoted over adipogenic differentiation even when cells were cultured under conditions favoring adipogenesis [24].

Recently, some studies have reported high expression of YAP1 in osteosarcoma specimens with subsequently a higher expression of target genes related to the Hippo pathway [13, 14, 25]. When YAP1 was knocked down by shRNA in MG-63 osteosarcoma cell line, proliferation and invasion were inhibited through inactivation of RUNX2 signaling [14]. Additionally, tumor growth was decreased following YAP suppression in murine xenografts [12] and in transgenic models [13]. High expression of YAP1 by immunohistochemistry in a series of biopsies of osteosarcomas compared to normal bone was reported by one study [14]. YAP1 expression was correlated with Enneking staging, with higher expression linked to stages II and III. Enneking staging is based on the tumor grade (low versus high), local extension and presence or absence of metastases. Based on this data, Zhang *et al.* showed higher YAP1 expression in high-grade osteosarcomas (conventional osteosarcomas) compared to low-grade osteosarcomas, which are known to have different oncogenic pathways. We showed in our study that among high-grade osteosarcomas, we could distinguish different subgroups of patients according to YAP/TAZ expression with different prognostic value. Albeit the number of patients was small, higher IRS was correlated to shorter OS in univariate analysis. Moreover, and for the first time, we reported that nuclear immunostaining of YAP/TAZ was an independent prognostic factor in multivariate analysis for PFS. Since YAP/TAZ proteins translocate to the nucleus and act as transcriptional co-activators on TEADs or on other transcriptional factors such as RUNX2, the detection of YAP/TAZ in the nucleus by immunohistochemistry suggests that transcriptional activity of target genes that induce cell proliferation is activated in most high-grade osteosarcomas and could in part explain the poor prognostic value of this marker for PFS. Interestingly, metastasis specimens had higher YAP/TAZ IRS compared to biopsy specimens which may indicate that YAP/TAZ expression is increased during the metastatic evolution of the disease. In our study, no statistical link between YAP/TAZ expression and response to preoperative chemotherapy was found while in other tumors such as oral squamous cell carcinoma YAP was shown to confer resistance to cisplatin [26]

Despite multiple evidences of the role of β1-integrin in tumor progression and cell proliferation [15], only few data are available concerning its expression in high-grade osteosarcomas yet. β1-integrin synthesis is increased following mechanical strain on human osteosarcoma TE-85 cells [27] and presence of β1-integrin on the membrane of osteosarcoma-derived osteoblasts (SAOS and MG63 cell lines) has been demonstrated by indirect immunofluorescence [16]. Recently, one study showed that the use of an anti-β1-integrin antibody inhibited the lung seeding of osteosarcoma cells [17] (143B human osteosarcoma cell line). In our study, we found that β1-integrin was widely expressed in conventional osteosarcomas. Additionally, membranous expression of β1-integrin by osteosarcoma was correlated with shorter overall survival. Albeit the small number of metastasis specimens, we found that β1-integrin membranous pattern of expression was always present in the metastatic samples, compared to 56% of the primitive tumors. The eventual correlation between β1-integrin membranous location and a worse prognosis is interesting since β1-integrin binds cells to their extracellular environment and thereby may also regulate signaling pathways [28].

Hippo pathway defects contribute to the development of cancer, but YAP and TAZ are also involved in regeneration following injury. The two sides of this pathway promoted great interest for the devellopement of molecules than can modulate the hippo pathway for anti-cancer treatment but also for regenerative medicine [29]. Verteportfin, a member of the porphyrin family, is used clinically as a photosensitizer for photodynamic therapy to treat choroidal neo-vascularization, especially in age-related macular degeneration [30]. Without light activation, Verteporfin selectively binds to YAP, changes its conformation thus inhibiting its interaction with TEAD2 and ultimately its transcriptional activity. Its interest for targeting YAP signaling during tumor progression have been further highlighted by the observation of a significant reduction of tumor burden in mice treated with Verteporfin [31]. Recently, TAZ and YAP were shown to be constitutively activated oncoproteins in sarcoma cell line and that Verteporfin decreased colony formation in soft agar and expression of Connective Tissue Growth Factor (CTGF) in sarcoma cell lines harboring activation of TAZ and YAP [32]. However, in this study no osteosarcoma cell line was investigated.

The effects of verteporfin on the bone micro-environement in primary bone tumors is not well known, as only bone metastases have been treated by Photodynamic therapy. However, the use of Verteporfin has been shown to ablate tumors and yield short-term improvements in vertebral architecture and biomechanical strength, in particular in combination with bisphosphonates [33]. Yet, no study has investigated the effects of Verteporfin on osteosarcoma cell lines selectively, in particular its anti-tumoral effect and its consequences on bone metabolism and osteoclastogenesis. The potential use of verteporfin to treat osteosarcoma could be a promising alternative therapeutic approach to convential chemotherapy, specifically for high-grade osteosarcoma with high expression of β1-integrin and YAP.

In conclusion, we have shown that YAP/TAZ and β1-integrin immunohistochemical expression in conventional osteosarcomas biopsies, performed before chemotherapy, is correlated in our study with bad prognosis. This study as the others published offers another convincing proofs that members of the Hippo pathway, namely YAP/TAZ and β1-integrin, could be potential new therapeutic targets: hippo pathway inhibitors such as verteporfin should be tested in this disease [31, 34].

## MATERIALS AND METHODS

### Immunochemistry

Automated immunohistochemistry was performed on four micrometers sections obtained from a tissue micro-array (TMA) comprising conventional osteosarcomas tumoral material. All tumor specimens were fixed in 4% formalin, mild decalcification with formic acid was applied when necessary. Specimens embedded in the TMA encompassed tissue material from 69 diagnostic biopsies and 19 specimens of pulmonary metastases from 69 patients with known clinical data, at Timone hospital (Marseille, France) from 1995 to 2012. Biopsies were performed prior to any chemotherapy. TMA construction was performed as previously described [19]: briefly, three representative areas were carefully selected on hematoxylin-eosin stained slides of the donor block, in order to punch core cylinders with a diameter of 1 mm that were secondly deposited inside two different TMA blocks (a reference block, and an identical twin block) using a specific arraying device (Alphelys). The immunohistochemistry was performed on a Ventana autostainer (Benchmark XT, Ventana Medical Systems SA, Illkirch, France) using antibodies anti-β1-integrin (Abcam, clone 4B7R, dilution 1/20, 32 min incubation) and anti-YAP/TAZ (Cell Signaling Technology, clone D24E4, dilution 1/25, 1 hour incubation), using heat retrieval procedure (1 hour at 98°C with buffer pH = 8). Melanoma and colon adenocarcinoma tissues were used as positive control for β1-integrin and for YAP/TAZ, respectively. Omission of primary antibody and irrelevant antibodies of the same isotype were used as negative controls. For each protein, immunohistochemical expression was semi-quantitatively assessed using immunoreactive score (IRS) as previously described [20, 21] and the cellular localization of the immunostaining (membranous, cytoplasmic or nuclear) was also noted. IRS can range from 0 to 12 and two classes were defined for the study: negative to weak staining (corresponding to IRS = 0-6) versus moderate to strong staining (corresponding to IRS = 7-12). Briefly, assessment of IRS is based on the proportion of stained cells which is scored from 0 to 4, multiplied by the intensity of staining, ranging from 0 to 3: no positive cells (0), < 10% of positive cells (1), 10-50% of positive cells (2), 51-80% of positive cells (3), > 80% of positive cells (4), no color reaction (0), mild reaction (1), moderate reaction (2), intense reaction (3) is used to calculate this score.

### Statistical analysis

Statistical analysis was performed using IBM SPSS Statistics software version 20 (IBM SPSS Inc., Chicago, IL, USA). Data were expressed as mean ± the standard deviation or median with interquartile range. Association between two categorical variables was assessed using the Chi square or Fisher's exact test. For continuous variables, the student t-test or Mann-Whitney U test was used to assess the association. Overall Survival (OS) and Progression-Free Survival (PFS) rates were estimated by the Kaplan-Meier method. OS was computed from the date of the death or last follow up. PFS was defined as time from the date of initial diagnosis to the date of progression or recurrence or last follow-up. Comparisons between survival curves were based on the log-rank test. A multivariate analysis was performed using Cox's model to estimate independent prognostic factors for OS and PFS. Hazard ratios with their 95% confidence interval were calculated. A two-sided P value of less than 0.05 was considered statistically significant.
